# Finite element analysis of the biomechanical behavior of four osseointegrated prosthetic designs

**DOI:** 10.3389/fbioe.2025.1694169

**Published:** 2025-12-10

**Authors:** Zi-Xuan Guo, Zhen Wang, Xue-Lin Zhao, Cheng-Wei Cao, Zi-Fu Huang, Zhao-Hang Sun, Yu-Shu Zheng, Meng Xu

**Affiliations:** Department of Orthopedics, The Forth Medical Center of PLA General Hospital, Beijing, China

**Keywords:** finite element, osseointegrated prosthesis, stress shielding, analysis, biomechanical

## Abstract

**Objective:**

Osseointegration is a critical determinant of the long-term stability and functional performance of orthopedic implants, with prosthetic morphology exerting substantial influence on biomechanical loading and bone–implant interface dynamics. This study aimed to evaluate the biomechanical behavior of four representative osseointegrated prosthetic designs using finite element analysis, to inform clinical application and guide optimization in prosthetic design.

**Methods:**

Three-dimensional finite element models were constructed to simulate host bone integrated with four distinct prosthetic configurations: (1) a threaded prosthesis representing the Osseointegrated Prostheses for the Rehabilitation of Amputees system, (2) a smooth press-fit prosthesis simulating the Osseointegrated Prosthetic Limb, (3) a titanium alloy prosthesis with a multi-porous surface, and (4) a molybdenum-rhenium (Mo-Re) alloy prosthesis with a multi-porous surface. Simulated physiological loading conditions were applied to evaluate stress distribution within prosthetic structures, interfacial mechanics at the bone-prosthesis junction, and stress transfer to surrounding osseous tissue.

**Results:**

All four prosthetic designs exhibited stress concentration at the distal stem region, with peak stress values ranging from 179 to 185 MPa, indicating comparable load-bearing characteristics. Incorporation of a multi-porous surface effectively reduced stress concentration on the inner cortical wall associated with groove geometry. The two multi-porous configurations demonstrated similar load transfer patterns, with maximum stress in adjacent bone tissue recorded at 20.4 MPa. The Mo–Re alloy prosthesis exhibited reduced deformation under equivalent loading due to its higher elastic modulus. Maximum stress within the porous section was 5.3 MPa for the Mo–Re prosthesis and 9.3 MPa for the titanium alloy variant, with no evidence of critical stress accumulation.

**Conclusion:**

The multi-porous Mo–Re alloy prosthesis demonstrated favorable mechanical compatibility through the optimized integration of material properties and structural design. Findings from the finite element analysis support its potential utility in osseointegrated orthopedic applications.

## 1 Introduction

Despite significant advances in modern medical care and interventional techniques, the global incidence of limb amputation remains substantial (Krueger et al., 2012). Contributing factors include population aging, road traffic injuries, regional conflicts, and acts of terrorism (Ephraim et al., 2003). Prosthetic limb systems are designed to enhance mobility, autonomy, safety, and overall quality of life among individuals with limb loss (Millstein et al., 1985). Conventional prosthetic designs most commonly employ a socket-type suspension system, in which a socket-type prosthesis externally interfaces with the residual limb. However, approximately one-third of prosthesis users experience chronic dermatological complications such as poor socket fit, excessive perspiration, and pressure ulcers, which can impair mobility and negatively impact quality of life (Lyon et al., 2000; Meulenbelt et al., 2009; Hagberg and Brånemark, 2001).

The concept of osseointegration, first introduced in the 1920s by Professor Brånemark (Brånemark et al., 1984), involves a direct structural and functional connection between a load-bearing implant and bone tissue, establishing skeletal anchorage without the interposition of fibrous tissue (Brånemark et al., 2001). Full contact between the intraosseous implant and cortical or trabecular bone is not required; successful osseointegration is generally characterized by direct contact with approximately 95% of the cortical bone surface and 50% of the prosthetic surface. Osseointegrated prosthetic systems offer multiple advantages, including direct control of the prosthetic limb, enhanced mechanical stability (Aschoff et al., 2010; Hagberg et al., 2008; Hagberg and Brånemark, 2009), improved fixation, increased comfort during seated postures, greater joint range of motion, reduced time for donning and doffing, improved proprioceptive feedback (Hagberg et al., 2005), and the phenomenon of osseoperception (Lundberg et al., 2011). These benefits collectively contribute to improved ambulatory function and enhanced quality of life (Frossard et al., 2008).

Extensive research, both in China and internationally, has focused on addressing the fundamental scientific and engineering challenges associated with implantable osseointegrated prostheses. Areas of focus include advancements in manufacturing technologies, micromorphological regulation, and the development of bioactive surface coatings (Li et al., 2016; Lewallen et al., 2015). Through iterative technological improvements and clinical validation, systems such as the Osseointegrated Prosthesis for the Rehabilitation of Amputees and the Osseointegrated Prosthetic Limb have reached a mature stage of development, enabling broad clinical adoption (Hagberg et al., 2014; Muderis et al., 2016; Al Muderis et al., 2017). However, unresolved challenges remain, including prosthetic loosening and periprosthetic bone resorption, both of which are linked to aberrant biomechanical interactions at the bone–prosthesis interface (Hagberg et al., 2020; Tsikandylakis et al., 2014). The local mechanical environment, particularly the magnitude and distribution of stress and strain, plays a critical role in modulating bone remodeling and the progression of the osseointegration process. Excessive localized stress may lead to prosthetic fatigue, while insufficient mechanical loading, or stress shielding, can result in bone resorption.

A major biomechanical limitation of metallic osseointegrated prostheses lies in the substantial mismatch between the elastic modulus of commonly used metal alloys and that of human bone. For instance, titanium alloys exhibit an elastic modulus of approximately 110 GPa, compared to 12 GPa for cortical bone and ∼100 MPa for cancellous bone. This disparity in stiffness contributes to uneven post-implantation load distribution, with high implant stiffness resulting in localized stress concentration within the prosthesis and insufficient mechanical stimulation of adjacent bone. This phenomenon, known as the stress-shielding effect, can lead to progressive bone resorption and may ultimately compromise implant stability and longevity.

To mitigate this issue, multi-porous prosthetic structures have emerged as a critical engineering solution. The introduction of controlled porosity into metallic implants reduces overall stiffness, thereby improving biomechanical compatibility with host bone (Sun et al., 2022). Porous architectures also promote cellular ingrowth and facilitate stable osseointegration—addressing the limitations of conventional solid metallic implants, which lack inherent capacity for direct bone integration (Wang et al., 2016). However, increased porosity also diminishes load-bearing capacity, presenting a fundamental design trade-off between reduced stiffness for biomechanical matching and sufficient structural strength to endure physiological loads.

Addressing this challenge requires the synergistic optimization of both material selection and pore architecture. By tailoring porosity characteristics and selecting high-performance metallic alloys, it is possible to enhance both mechanical integrity and biological performance. Recent studies have demonstrated that molybdenum–rhenium (Mo–Re) alloys offer notable advantages in this context (Mok et al., 2023). When integrated into multi-porous designs, Mo–Re alloys exhibit stiffness values comparable to both cortical and cancellous bone, thereby reducing stress shielding. Their high tensile strength (1000–1500 MPa) offsets porosity-induced reductions in structural integrity. Additionally, Mo–Re alloys display excellent corrosion resistance in physiological environments by forming a stable passive oxide layer, contributing to long-term durability post-implantation.

Despite these promising properties, no finite element studies to date have systematically compared Mo–Re alloy-based multi-porous prosthetic designs with other commonly used configurations in terms of stress distribution at the transfemoral bone–implant interface. This represents a significant gap in the current understanding of how prosthetic design influences mechanical compatibility in transfemoral osseointegration.

In the present study, a finite element model simulating a transfemoral amputation was developed to evaluate the biomechanical behavior of four distinct prosthetic designs. Stress distribution patterns within the femur and at the bone–prosthesis interface were assessed. The objective was to generate quantitative biomechanical evidence to inform prosthetic design optimization and support improved clinical outcomes in osseointegrated limb reconstruction.

## 2 Materials and methods

### 2.1 Model development

**FIGURE 1:**
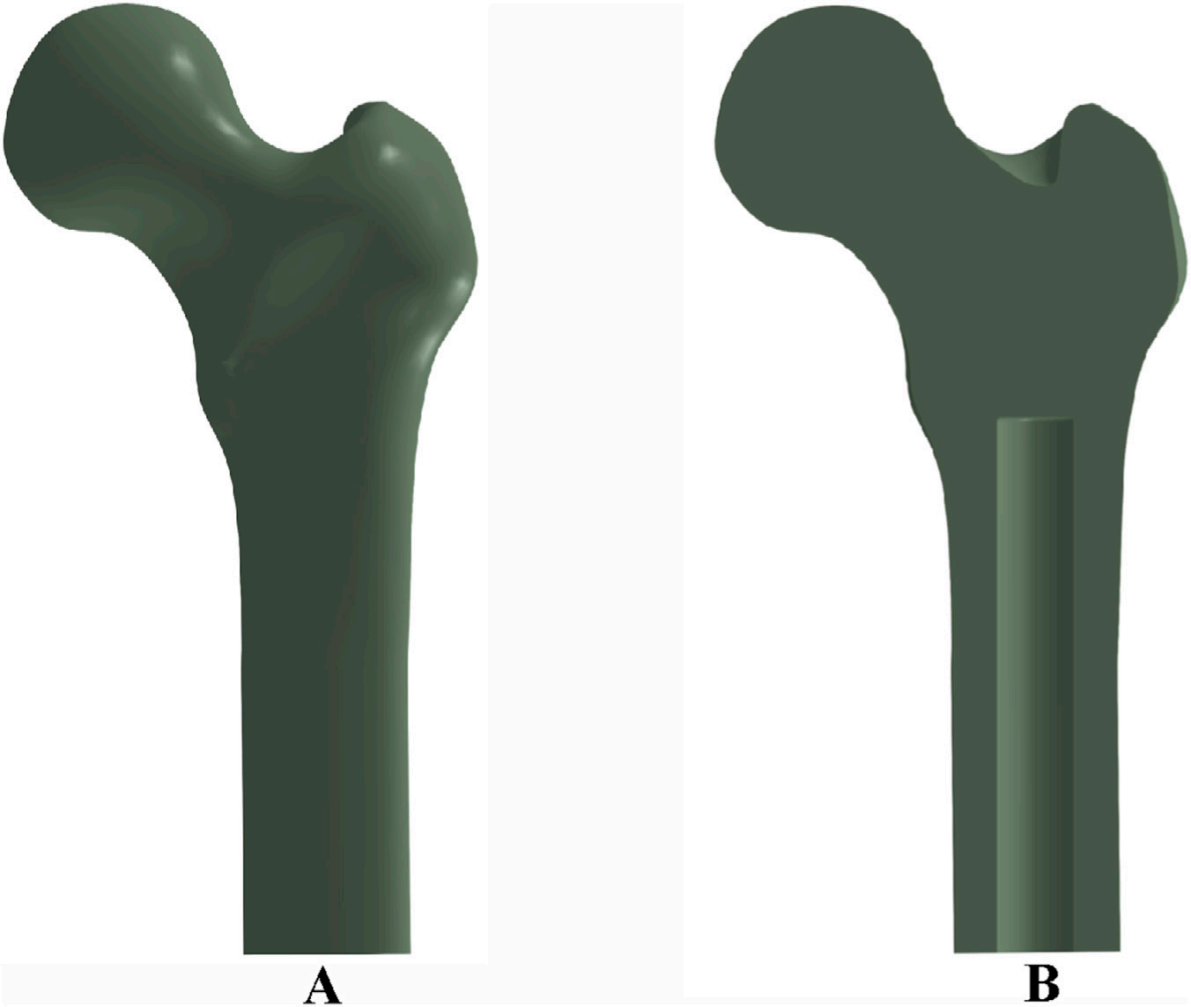
Femoral bone model and implant cavity configuration: **(A)** three-dimensional reconstruction of the femur; **(B)** cross-sectional view showing the medullary cavity and implant cavity dimensions.

Two-dimensional computed tomography (CT) images of a human femur were acquired and imported into the three-dimensional image processing software Avizo 9.0 for anatomical reconstruction. The reconstructed femoral geometry was exported as a surface mesh in STL (.stl) format. These files were subsequently imported into Rhino 7.0 software to generate solid models suitable for finite element analysis. The final three-dimensional solid reconstruction of the femur is shown in Figure 1A.

To simulate prosthesis implantation, an intramedullary cavity was defined within the femoral canal, measuring 140 mm in length and 22 mm in diameter, as depicted in Figure 1B.

### 2.2 Material properties and prosthesis design

**FIGURE 2:**
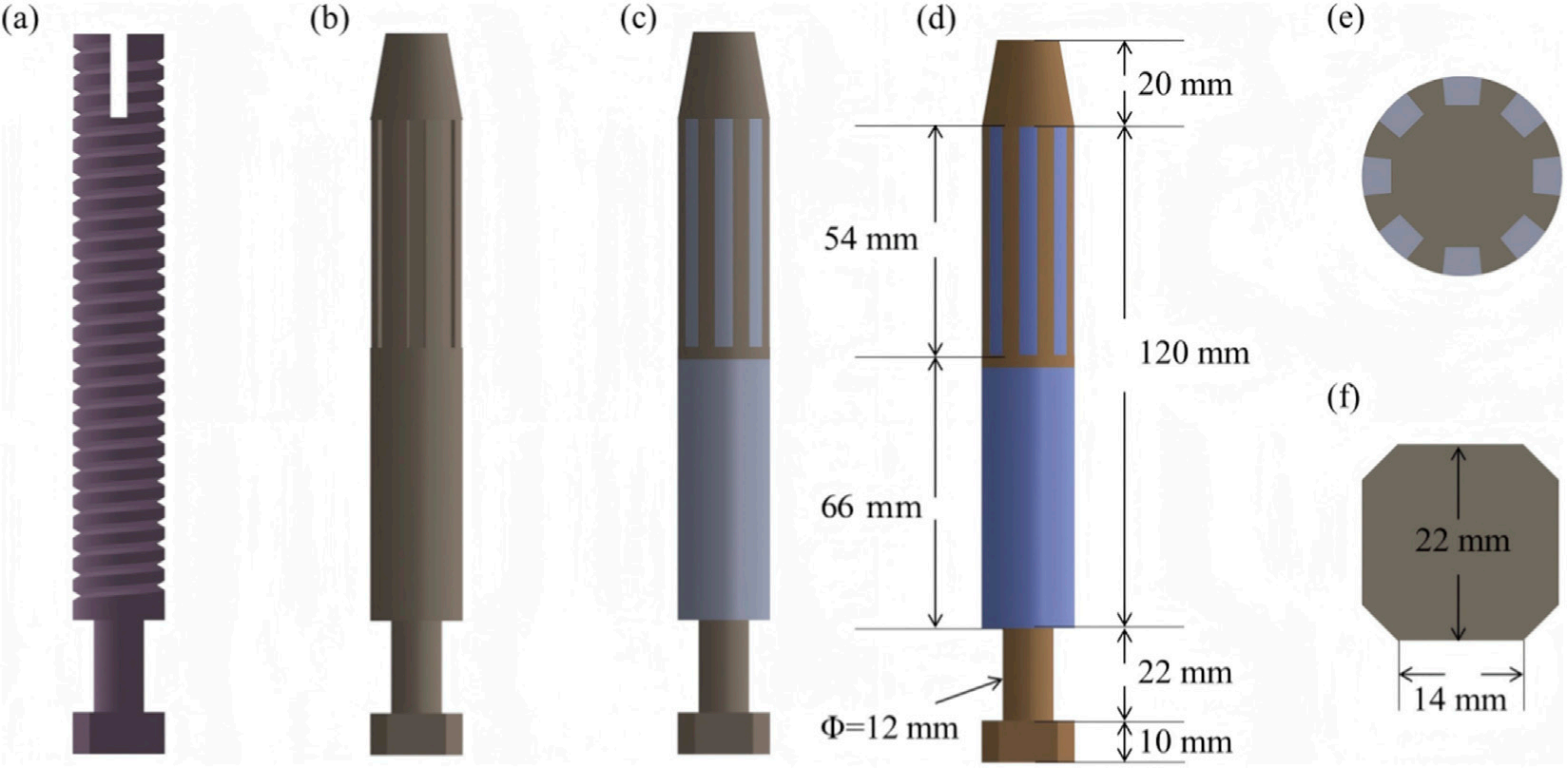
Design features and dimensional parameters of the four prosthetic models: **(a)** threaded titanium alloy prosthesis; **(b)** grooved titanium alloy prosthesis; **(c)** titanium alloy prosthesis with multi-porous coating; **(d)** molybdenum–rhenium (Mo-Re) prosthesis; **(e)** cross-sectional view showing groove array and internal structure; **(f)** outer end design incorporating an octagonal interface for external component fixation.

Four osseointegrated prosthetic designs were developed for this study, as illustrated in Figure 2. The first design, a threaded titanium alloy (Ti-6Al-4V) prosthesis, is shown in Figure 2a. The second design, depicted in Figure 2b, incorporates surface grooves intended to facilitate interfacial friction and material exchange. Both designs include full-length grooves at the distal end of the prosthesis, filled with a multi-porous structure characterized by a porosity of 49% and an average pore size of 400 μm (Frosch et al., 2021). This configuration is intended to enhance frictional engagement and support osseointegration through bone ingrowth.

The third design (Figure 2c) adopts a similar structure to the second but features a continuous porous surface layer. All three prostheses (Models 1–3) were fabricated entirely from Ti-6Al-4V alloy. In contrast, the fourth prosthesis comprises a Mo–Re alloy core for the solid region, while the porous region is composed of commercially pure titanium. The selection of Mo–Re alloy was based on its high elastic modulus, favorable radiological attenuation, and low magnetic susceptibility, characteristics that render it suitable for long-term biomedical implantation. The geometry of the solid and porous regions in the fourth model mirrors that of the third prosthesis, differing only in the use of Mo–Re alloy in the solid segment.

Dimensional specifications for each prosthesis are provided in Figure 2. Eight circumferential grooves were arranged in a circular array, as shown in Figure 2e, and an octagonal cross-sectional geometry was applied at the distal prosthetic end to enhance mechanical coupling with external prosthetic components.

**FIGURE 3:**
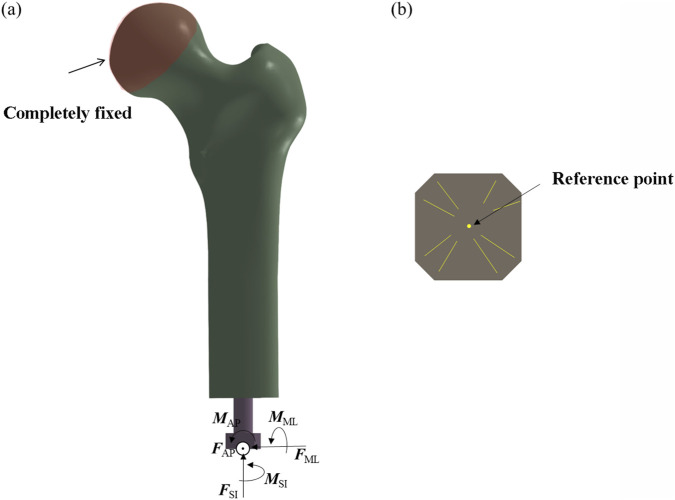
Finite element model showing applied boundary conditions and loading scheme. **(a)** Fully fix the femoral head. **(b)** Take the center as the reference point.

The applied boundary conditions and loading scheme are illustrated in Figure 3. The acetabular region of the femur was fully constrained to simulate anatomical fixation. Concentrated loads and moments were applied via a reference point rigidly coupled to the distal prosthesis end, replicating physiological loading conditions. For the threaded prosthesis (Model 1), a frictional contact interface was defined between the thread surface and the medullary canal wall, representing the immediate postoperative state. Similarly, Model 2 employed frictional contact between the grooved surface and the inner cortical wall.

For the third and fourth prosthetic models, the porous regions were defined as tied contact interfaces with the inner wall of the medullary canal to simulate mechanical fixation following complete osseointegration. In contrast, the smooth regions of these prostheses were assigned frictional contact interfaces. A uniform coefficient of friction of 0.1 was applied across all frictional contact regions (Gao et al., 2019).

This tied contact condition replicates the biomechanical behavior observed after successful osseointegration, wherein bone tissue infiltrates the porous architecture of the implant, forming a biological bond. At this stage, relative micromotion between the implant and host bone is eliminated, enabling direct transmission of mechanical loads across the bone–implant interface. This setup is consistent with the mechanical characteristics associated with the clinically defined state of complete osseointegration.

To ensure consistent comparison with the threaded and smooth-surfaced titanium alloy prostheses, the smooth regions of the porous-structured prostheses were also modeled using frictional contact with the inner wall of the medullary canal (Li and Felländer-Tsai, 2021). This standardized contact condition enables consistent boundary conditions across all models, isolating the effects of porous architecture and material composition.

By differentiating the contact definitions—tied contact for porous regions (to represent post-osseointegration fixation) and frictional contact for smooth regions—while maintaining uniform frictional behavior in non-porous areas, this modeling approach eliminates confounding variables. Consequently, any differences in biomechanical performance among the four prosthetic designs can be attributed specifically to the presence or absence of porous structures and to differences in material properties. This methodological consistency enhances the scientific validity and reliability of the comparative finite element analysis.

The titanium alloy (Ti-6Al-4V) was assigned an elastic modulus of 110 GPa, a density of approximately 4.43 g/cm^3^, and a yield strength in the range of 900–1100 MPa. The Mo–Re alloy was modeled with an elastic modulus of 350 GPa, a density of approximately 13 g/cm^3^, and a yield strength ranging from 1200 to 1600 MPa. The porous structure was defined with an elastic modulus of 5 GPa (Wen et al., 2001). For all simulations, the porous region was assumed to exhibit isotropic mechanical behavior, with uniform properties in all spatial directions. This assumption supports the accuracy and consistency of the material property parameters applied in the finite element analysis. Further details are provided in Supplementary Table S1.

### 2.3 Loading conditions

Two critical phases of the human gait cycle—heel strike and toe-off—were selected to represent physiological loading conditions applied to the femur. These phases correspond to periods of high mechanical demand and are associated with significant strain distributions in the femoral structure, as demonstrated in prior biomechanical studies (Robinson et al., 2020). To reflect realistic load scenarios, simulation inputs were based on average *in vivo* measurements reported in the literature and correspond to approximately 25% (heel strike) and 55% (toe-off) of the gait cycle (Lee et al., 2007; Frossard et al., 2010). Load parameters are summarized in Table 1.

**TABLE 1:** Loading parameters applied in the finite element simulations at 25% (heel strike) and 55% (toe-off) phases of the gait cycle.

Load case	Load					
	FSI [N]	FAP [N]	FML [N]	MSI [Nm]	MAP [Nm]	MML [Nm]
1	780	100	−20	−2.0	30.8	−7.2
2	180	120	40	0.0	37.3	4.1

FSI, is the axial force; FAP, is the front and rear force; FML, is the internal and external force; MSI, is the axial torque; MAP, is the front and rear torque and MML, is the internal and external torque.

During the heel strike phase (Load Case 1), the applied force vectors along the anatomical coordinate system included 780 N in the superior–inferior (SI) direction, 100 N in the anterior–posterior (AP) direction, and −20 N in the medial–lateral (ML) direction, where the negative sign indicates a direction opposite to the positive axis definition. The corresponding applied moments were −2.0 N·m in the SI direction, 30.8 N·m in the AP direction, and −7.2 N·m in the ML direction.

In the toe-off phase (Load Case 2), the applied forces were 180 N in the SI direction, 120 N in the AP direction, and 40 N in the ML direction. The associated moments were 0.0 N·m in the SI direction, 37.3 N·m in the AP direction, and 4.1 N·m in the ML direction.

Each directional component corresponds to a distinct biomechanical function. The superior–inferior force (FSI) acts along the body’s longitudinal axis and corresponds to gravitational force and the vertical component of the ground reaction force. During the stance phase, the upward ground reaction force interacts with body weight to influence loading in the femur and other lower limb bones.

The anterior–posterior force (FAP), acting along the sagittal plane, reflects forward–backward motion and is associated with propulsion during toe-off and braking during heel strike. This component captures anterior–posterior loading conditions in the lower limb.

The medial–lateral force (FML), acting along the coronal plane, contributes to mediolateral stability and balance control during gait.

The superior–inferior moment (MSI) causes rotation about an axis perpendicular to the SI direction and affects femoral motion in the sagittal plane, particularly during flexion and extension movements.

The anterior–posterior moment (MAP) results in rotation around an axis along the AP direction and is related to internal and external rotation of the femur, contributing to rotational posture regulation.

The medial–lateral moment (MML) induces rotation around an axis aligned with the ML direction and influences flexion and extension movements, affecting overall mechanical balance during gait.

### 2.4 Evaluation indices

The mechanical performance of the bone–prosthesis interface was evaluated using three key parameters: peak contact stress, contact area ratio, and interfacial sliding displacement.

The response of the surrounding bone tissue was assessed using two metrics: the maximum von Mises stress and the stress shielding ratio (SSR). The SSR quantifies the reduction in mechanical stimulus experienced by cancellous bone following prosthesis implantation and was calculated as follows:

SSR = 1 - (stress of cancellous bone with prosthesis/stress of cancellous bone without prosthesis) × 100%

Prosthetic structural performance was evaluated based on the maximum von Mises stress within the implant and the stress concentration factor (SCF), which was defined as:

SCF = maximum stress/average stress.

## 3 Results

### 3.1 Overall stress distribution in the bone-prosthesis system

**FIGURE 4:**
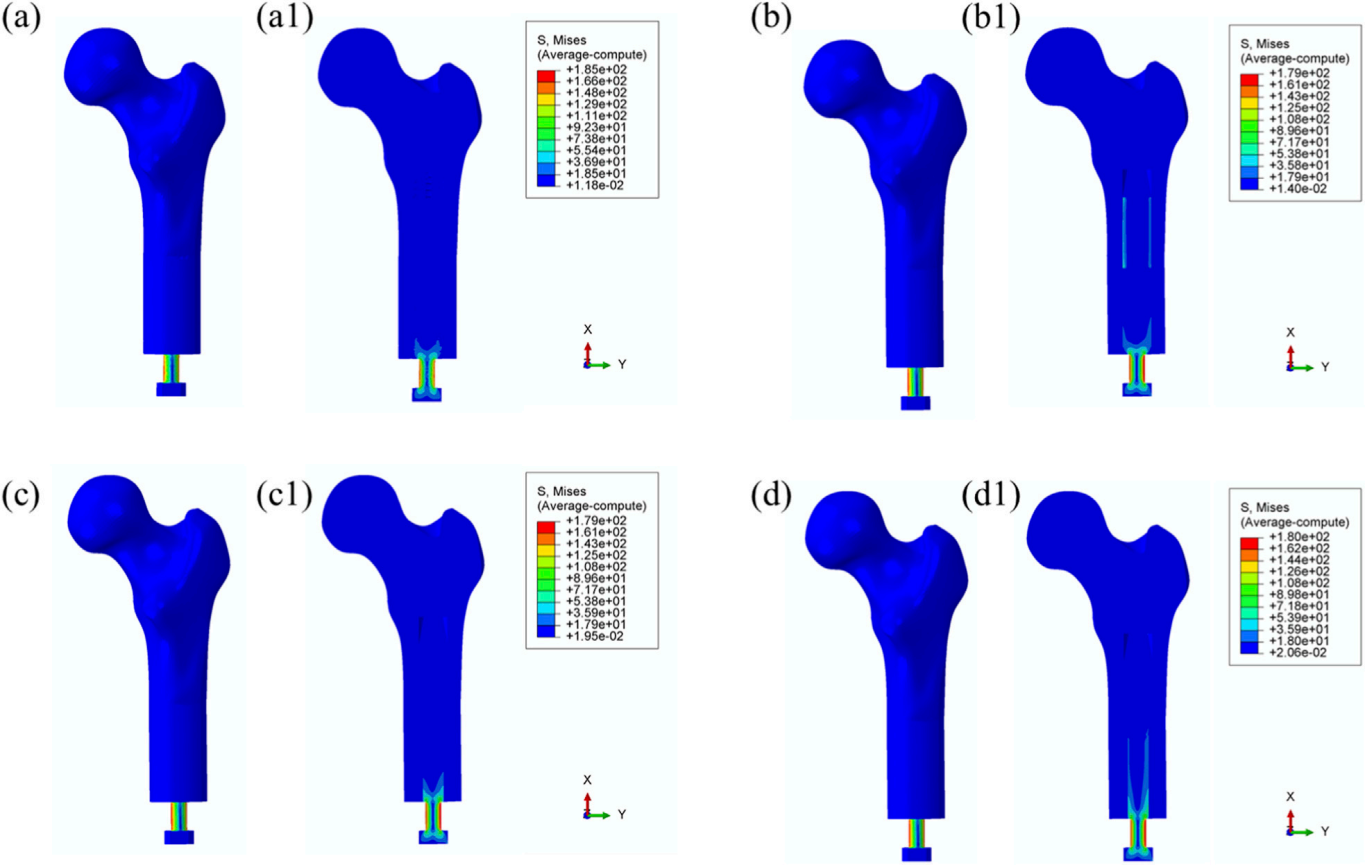
Overall von Mises stress distribution in the bone–prosthesis system under 25% gait cycle loading (heel strike) for each prosthesis type. **(a)** Threaded prosthesis; **(b)** Grooved prosthesis; **(c)** Titanium alloy prosthesis with multi-porous surface; **(d)** Mo-Re alloy prosthesis with multi-porous surface. **(a1–d1)** respectively denote the cross-sectional stress distribution of different osseointegrated prosthesis designs.

Figure 4 illustrates the von Mises stress distribution in the bone–prosthesis system under the 25% gait cycle loading condition (heel strike) for each of the four prosthetic designs. Across all models, the highest stress concentrations were observed at the outer stem region of the prostheses.

The maximum von Mises stress was recorded in the threaded prosthesis model, reaching 185 MPa. This elevated stress was primarily attributed to the combined effects of triaxial loading and applied bending moments on the prosthetic stem, resulting in localized stress accumulation. In comparison, the maximum von Mises stress values for the second, third, and fourth prosthetic designs were 179 MPa, 179 MPa, and 180 MPa, respectively, slightly lower than that observed with the threaded design.

The modest increase in stress in the threaded prosthesis may be associated with its geometric configuration, which likely resulted in a slightly longer effective moment arm under load, thereby contributing to higher localized stress. Relative to the threaded prosthesis (185 MPa), the stress reductions for the second, third, and fourth prostheses were calculated as 3.24%, 3.24%, and 2.70%, respectively.

**FIGURE 5:**
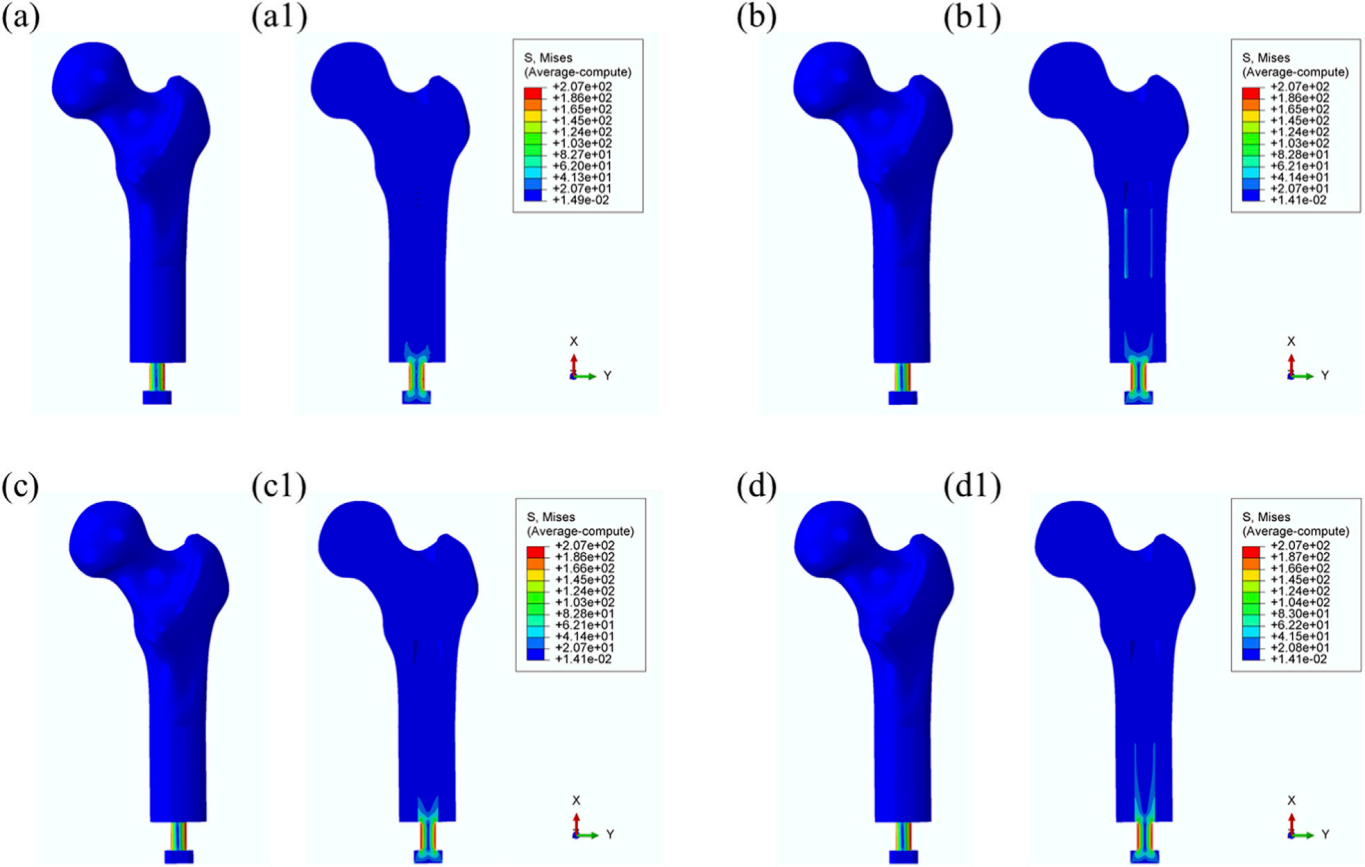
Overall von Mises stress distribution in the bone–prosthesis system under 55% gait cycle loading (toe-off) for each prosthesis types. **(a**) Threaded prosthesis; **(b)** Grooved prosthesis; **(c)** Titanium alloy prosthesis with multi-porous surface; **(d)** Mo-Re alloy prosthesis with multi-porous surface. **(a1–d1)** respectively denote the cross-sectional stress distribution of different osseointegrated prosthesis designs.

Similar stress distribution patterns were observed during the toe-off phase of the gait cycle (55%), as shown in Figure 5.

### 3.2 Bone tissue response

**FIGURE 6:**
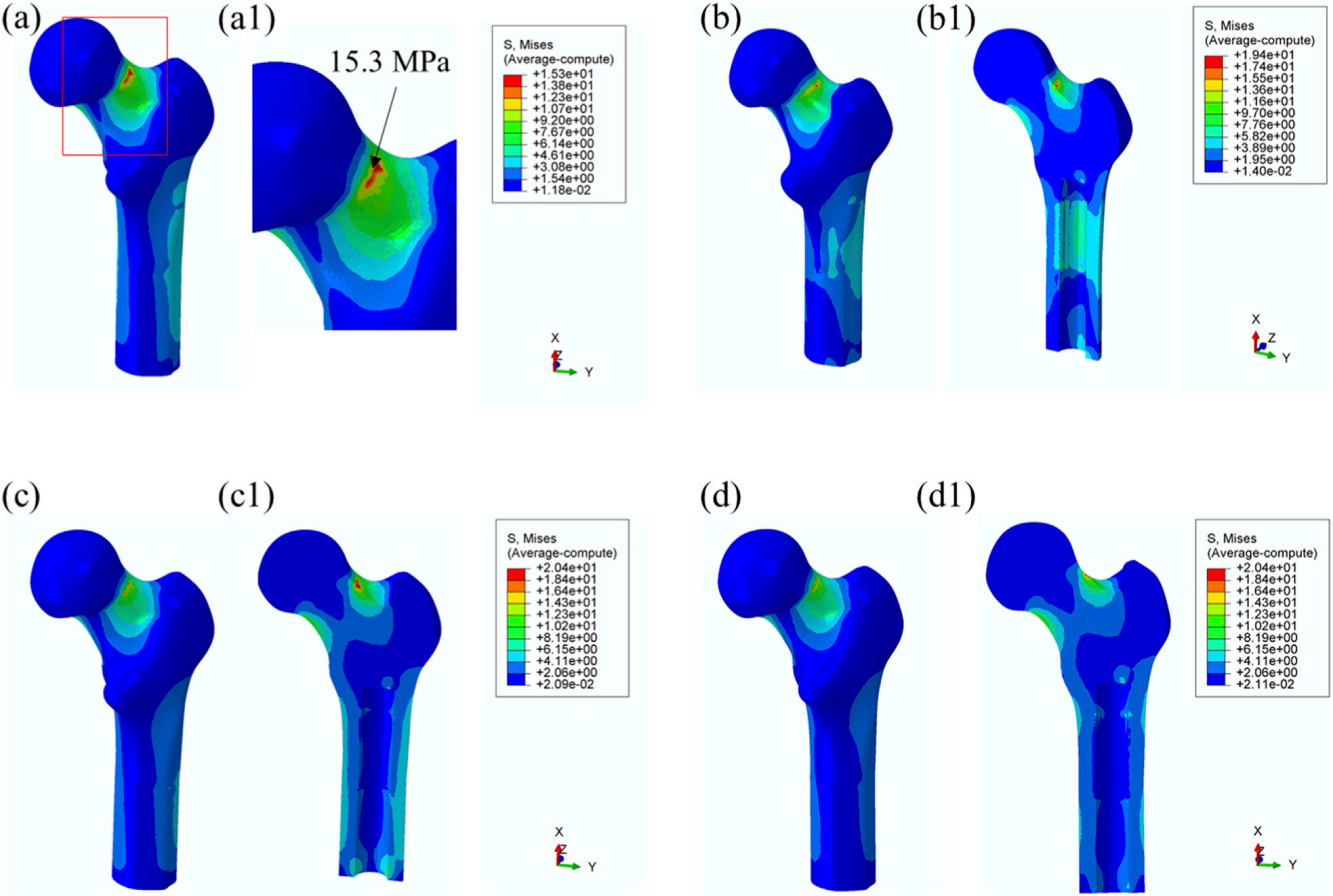
Von Mises stress distribution in surrounding bone tissue under 25% load (heel strike) with each prosthesis design. **(a) **Threaded prosthesis; **(b)** Grooved prosthesis; **(c)** Titanium alloy prosthesis with multi-porous surface; **(d)** Mo-Re alloy prosthesis with multi-porous surface. **(a1–d1)** respectively denote the cross-sectional stress distribution of different osseointegrated prosthesis designs.

Maximum von Mises Stress: Load transfer from the prosthesis to the surrounding bone is essential for preserving biomechanical function and contributes to localized stress concentrations within the bone tissue. As shown in Figure 6, no significant stress concentration was observed within the medullary cavity for most models.

In the model incorporating the threaded prosthesis, the highest stress concentration reached 15.3 MPa and was localized at the notch of the femoral neck, as illustrated in Figures 6a, 6(a1). In the second prosthesis model, the maximum von Mises stress increased to 19.4 MPa, as shown in Figure 6b. Additionally, this model exhibited stress concentrations exceeding 9 MPa within the medullary cavity, primarily due to the presence of a notch. These elevated stress levels were considered unfavorable for osseointegration at the bone–prosthesis interface and may contribute to an increased risk of localized bone resorption or fracture.

In contrast, the third and fourth prosthesis models, both incorporating porous structures, exhibited maximum von Mises stress values of 20.4 MPa post-implantation, as presented in Figures 6c,d. Although these stress levels were comparable to those observed in the second prosthesis, the presence of porous surfaces facilitated more uniform stress distribution and improved load transfer characteristics. Notably, the porous configurations effectively mitigated excessive stress accumulation within the medullary cavity, indicating a potential advantage in supporting osseointegration and enhancing clinical performance.

**FIGURE 7:**
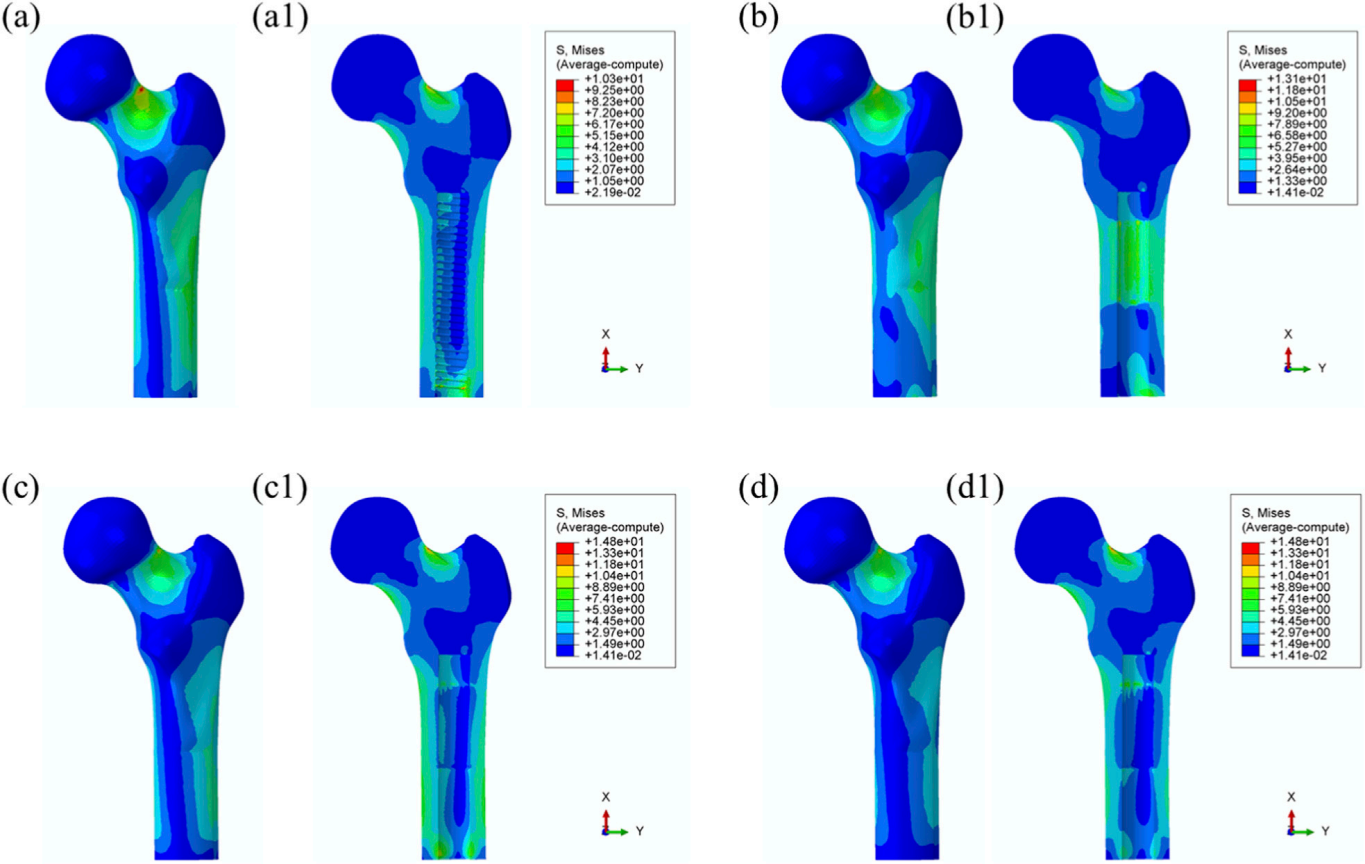
Von Mises stress distribution in surrounding bone tissue under 55% load (toe-off) with each prosthesis design. **(a)** Threaded prosthesis; **(b)** Grooved prosthesis; **(c)** Titanium alloy prosthesis with multi-porous surface; **(d)** Mo-Re alloy prosthesis with multi-porous surface. **(a1–d1)** respectively denote the cross-sectional stress distribution of different osseointegrated prosthesis designs.

Using the maximum stress value observed in the threaded prosthesis model (15.3 MPa) as the reference, the normalized maximum stress values for Models 2, 3, and 4 were 1.268, 1.333, and 1.333, respectively. These values indicate that the overall stress levels in the latter three models were moderately elevated compared to the threaded design. Comparable stress distribution patterns were also observed during the toe-off phase of the gait cycle, as shown in Figure 7.

### 3.3 Prosthetic performance

**FIGURE 8:**
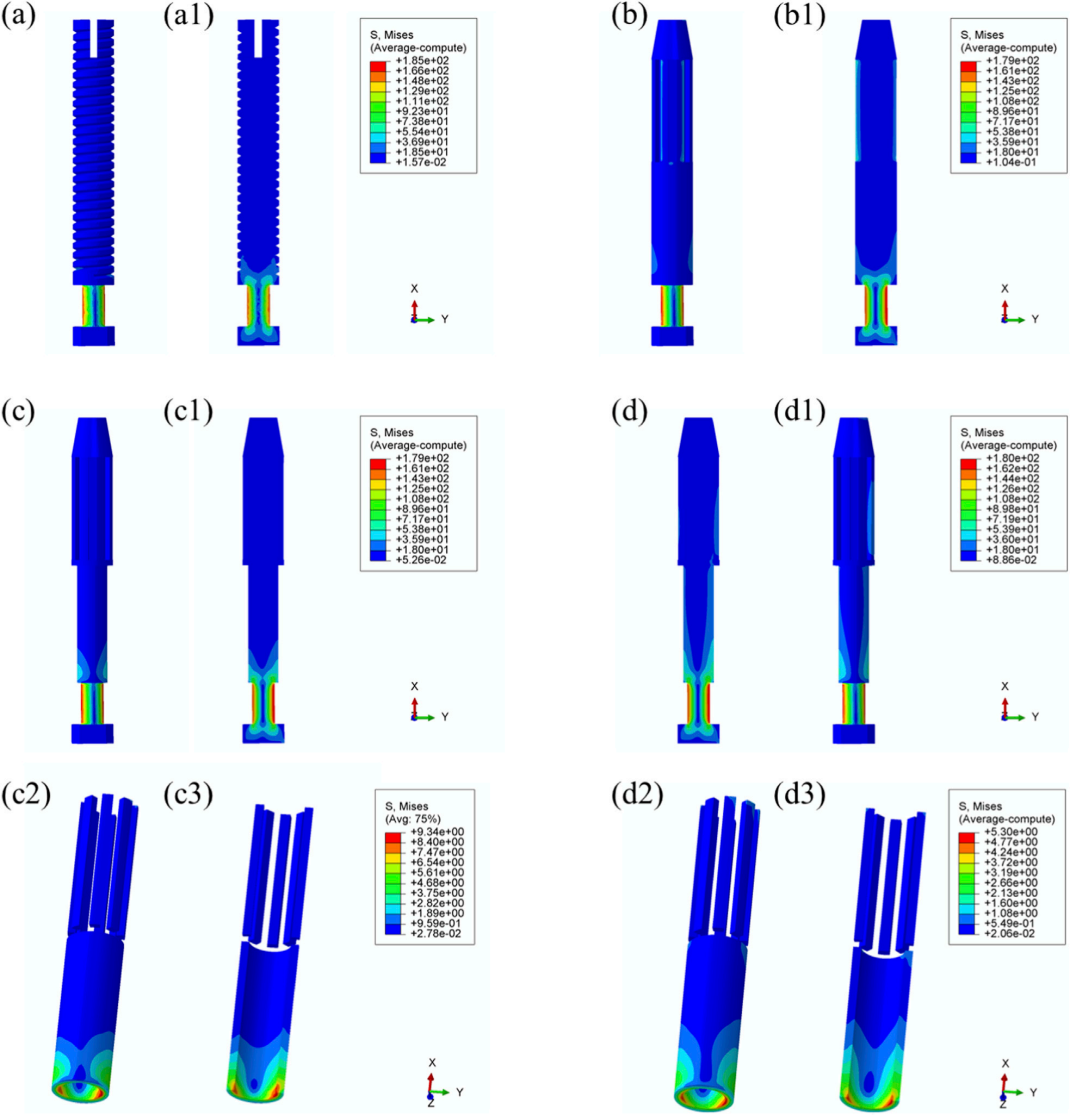
Von Mises stress distribution within the prostheses under 25% load (heel strike): **(a)** threaded prosthesis; **(b)** grooved prosthesis; **(c)** titanium alloy prosthesis with multi-porous surface; **(d)** Mo-Re alloy prosthesis; **(c2, c3, d2, d3)** stress distribution in the porous regions of the respective designs.

Maximum von Mises stress: The stress distribution characteristics of the four prosthetic designs were further evaluated, as illustrated in Figure 8. The maximum von Mises stress values for the threaded, grooved, titanium porous, and Mo–Re porous prostheses were 185 MPa, 179 MPa, 179 MPa, and 180 MPa, respectively—consistent with the values previously observed in the overall bone–prosthesis system.

In the threaded prosthesis model, stress concentration was primarily localized at the outer surface of the prosthetic stem end, extending inward, as shown in Figure 8a. A similar pattern was observed in the grooved prosthesis (Figure 8b), where the highest stress was concentrated at the stem end. Additionally, localized stress concentrations ranging from 35 to 53 MPa were detected at the groove sites. These areas corresponded spatially with stress concentrations on the inner wall of the medullary cavity and were likely attributable to the combined effects of interfacial friction and mechanical loading.

As illustrated in Figure 6, the incorporation of a multi-porous structure within the grooves effectively eliminated these localized stress concentrations on the inner wall of the medullary cavity. This effect was further supported by the stress distribution patterns observed in the third and fourth prosthesis models (Figures 8c,d), where the porous structures contributed to more uniform load distribution and mitigated stress peaks around the grooves.

In both the titanium and Mo–Re porous prostheses, von Mises stress extended from the porous region into the inner surface of the solid core. This stress transfer was attributed to the lower elastic modulus of the porous outer layer, which permitted greater deformation under load and consequently transmitted load to the higher-stiffness solid core. Figures 8c2, 8c3, 8d2, 8d3 display the stress distribution within the porous sections. In both models, stress concentrations were noted near the prosthetic stem end, aligning with regions of elevated stress in the adjacent solid components.

The maximum stress within the porous region of the titanium alloy prosthesis was 9.3 MPa, while the Mo–Re alloy prosthesis exhibited a lower value of 5.3 MPa. These stress concentrations reflect anisotropy in load distribution, resulting from the mechanical interaction between the porous and solid regions.

To quantify this difference, the stress reduction in the porous portion of Model 4 (Mo–Re alloy) relative to Model 3 (Ti–6Al–4V alloy) was calculated. The maximum stress of 5.3 MPa in Model 4 represents a 43.01% decrease compared to 9.3 MPa in Model 3, yielding a normalized value of 0.570. This result demonstrates a synergistic effect between the high elastic modulus of the Mo–Re alloy and the porous design. The rigidity of the Mo–Re solid core facilitates load dispersion, significantly reducing the stress burden on the porous region and thereby enhancing the mechanical performance of the composite structure.

**FIGURE 9:**
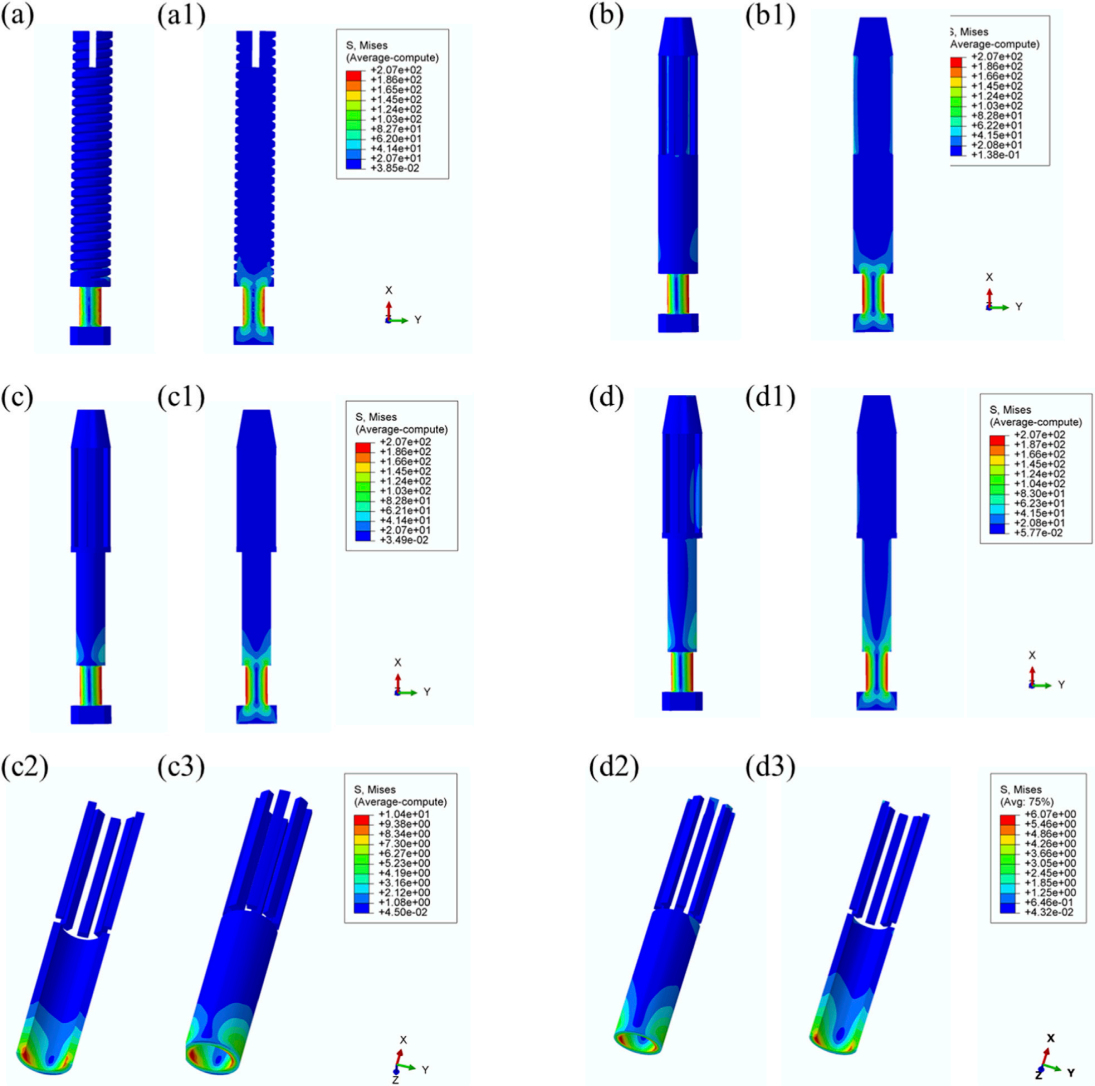
Von Mises stress distribution within the prostheses under 55% load (toe-off) across all designs. **(a)** Threaded prosthesis; **(b)** Grooved prosthesis; **(c)** Titanium alloy prosthesis with multi-porous surface; **(d)** Mo-Re alloy prosthesis; **(a1–d1)** respectively denote the cross-sectional stress distribution of different osseointegrated prosthesis designs. **(c2,c3,d2,d3)** stress distribution in the porous regions of the respective designs.

Comparable stress distribution patterns were observed under the toe-off loading condition (55% gait cycle), as illustrated in Figure 9. Additional data are provided in Supplementary Table S2.

## 4 Discussion

This study employed finite element analysis to evaluate the stress distribution characteristics of four transfemoral prosthetic designs under physiological loading conditions corresponding to the 25% (heel strike) and 55% (toe-off) phases of the gait cycle. The findings provide quantitative data to inform the optimization of prosthetic geometry and material selection, demonstrating that structural design parameters and material properties significantly influence interfacial mechanical behavior. These insights have direct implications for the development and clinical application of osseointegrated prosthetic systems.

Across all four prosthetic models, stress concentrations were consistently observed at the distal stem region, with maximum von Mises stress values ranging from 179 to 185 MPa. This pattern is attributable to the commonality in load transfer pathways, wherein the distal stem functions as the primary conduit for axial load transmission from the prosthesis to the femoral medullary canal. During the heel strike phase of the gait cycle, this region is subjected to approximately 25% of the vertical body load.

The observed uniformity in stress distribution across the prosthetic designs may also reflect similarities in morphological parameters, such as stem diameter and implantation depth. These results underscore the importance of optimizing load transfer at the distal stem interface. Design modifications that enhance conformity between the prosthetic stem and the medullary canal—such as the incorporation of a tapered stem geometry—may reduce localized stress concentrations while maintaining the mechanical stability of the implant.

Additionally, achieving more uniform contact pressure along the stem–bone interface may facilitate stress homogenization and reduce the risk of mechanically induced complications, including stress shielding and prosthetic loosening.

Notably, the incorporation of porous structures into grooved prostheses markedly reduced stress concentrations along the inner wall of the medullary cavity. This benefit arises from distinct differences in interfacial stress regulation mechanisms. While grooved designs enhance initial fixation through mechanical interlocking—a form of physical engagement achieved via the interaction between the prosthetic grooves and surrounding bone tissue, which restricts relative micromotion at the bone–prosthesis interface—the geometric discontinuities at the groove edges can induce local stress singularities. These focal stress elevations may increase the long-term risk of bone resorption and prosthetic loosening (Hagberg et al., 2020).

In contrast, multi-porous structures promote more uniform stress distribution via their three-dimensional, interconnected pore architecture. These structures enable bone tissue ingrowth, establishing a biomechanical interlock that facilitates stable and evenly distributed load transfer from the prosthesis to the host bone. Moreover, the stiffness of multi-porous regions can be modulated through porosity adjustment, allowing the elastic modulus of the implant surface to approximate that of adjacent bone tissue—particularly cancellous bone, which typically exhibits an elastic modulus on the order of 100 MPa. This design strategy mitigates both stress shielding and stress concentration by improving biomechanical compatibility.

These findings are consistent with prior research on porous metallic implants and further underscore the critical role of multi-porous structures in enhancing mechanical integration at the bone–implant interface.

In the present study, both porous prosthesis models demonstrated comparable load transfer characteristics, with the surrounding bone exhibiting maximum von Mises stress values of 20.4 MPa. These stress levels fall within the physiological tolerance range often described as the “favorable stress window” in bone remodeling theory, which proposes that mechanical stimuli in the range of 10–30 MPa promote osteoblast activity and extracellular matrix deposition. In contrast, excessive stress levels (>50 MPa) may result in microstructural bone damage, whereas insufficient mechanical loading (<1–2 MPa) is associated with bone resorption (Brinlee et al., 2022).

The stress magnitudes observed in this analysis suggest that the porous prosthetic designs provided adequate mechanical stimulation to facilitate osseointegration without exceeding thresholds that could compromise bone integrity. These findings offer a quantitative reference for the optimization of key design parameters in multi-porous structures, particularly porosity and pore size, to ensure effective load transfer and biologically favorable stress distribution.

Moreover, the comparable mechanical performance observed between the two porous prosthesis types indicates that, when fundamental design parameters are appropriately configured, specifically, porosity in the range of 50%–70% and pore diameters between 200 and 500 μm, multi-porous prosthetic designs can consistently deliver stable, physiologically compatible load transmission to the surrounding bone (Wang et al., 2016; Karageorgiou and Kaplan, 2005; Liu et al., 2015; Salou et al., 2015; Wang et al., 2023).

Compared to titanium alloy prostheses, the Mo–Re alloy counterparts exhibited reduced deformation under equivalent loading conditions. Specifically, the maximum von Mises stress in the porous section of the Mo–Re prosthesis was 5.3 MPa, compared to 9.3 MPa in the titanium alloy model. This finding underscores the synergistic potential of combining high-performance metallic materials with optimized porous architectures. Although Mo–Re alloys possess a high elastic modulus—which is typically associated with an increased risk of stress shielding—their high tensile strength (ranging from 1000 to 1500 MPa) effectively compensates for the mechanical strength reductions typically introduced by porous structures. Concurrently, the incorporation of a multi-porous design reduces the overall stiffness of the prosthesis, thereby mitigating the adverse effects commonly associated with high-modulus materials.

The observed reduction in stress within the porous region also contributes to improved mechanical stability by lowering the risk of fatigue failure under long-term cyclic loading conditions (Wang et al., 2023). These results support the application of high-modulus metals such as Mo–Re in osseointegrated prosthetic systems, provided that their structural architecture is appropriately optimized to balance material stiffness and mechanical compatibility.

To facilitate a clear comparison of the four transfemoral prosthesis designs—grooved, traditional solid, titanium alloy porous, and Mo–Re alloy porous—a summary is provided in Supplementary Table S3. The table presents key distinctions across structural features, material properties, mechanical performance metrics, and design implications.

The comparison indicates that the core differences among the four prosthesis types lie in the combination of structure (porous vs. non-porous) and material (conventional titanium alloy vs. Mo–Re alloy). These design variables directly influence mechanical behavior and, consequently, clinical applicability. Non-porous designs, such as the grooved and traditional solid prostheses, offer benefits in terms of initial fixation and structural simplicity; however, they are associated with inherent limitations, including localized stress concentration and stress shielding, which may compromise long-term osseointegration.

By contrast, porous prostheses mitigate these issues through biomechanical interlocking and stress dispersion. Among them, the Mo–Re alloy porous prosthesis demonstrates superior performance in stress distribution and deformation control, due to the combined effect of high elastic modulus and optimized porous architecture. This makes it particularly suitable for individuals with above-knee amputations who are subject to long-term cyclic loading.

These findings suggest a clear direction for prosthesis optimization: porous structures should be prioritized to support osseointegration, while high-strength materials—such as Mo–Re alloy—can be employed to counteract the mechanical limitations introduced by porosity. This strategy enables the dual goals of biomechanical compatibility and structural reliability, offering practical guidance for clinical prosthesis selection and future design refinement.

In this study, the feasibility of Mo–Re alloy prostheses incorporating multi-porous structures was preliminarily assessed using finite element simulations. While the simulation results provide valuable insights into the mechanical performance of these designs, several limitations must be acknowledged, and further investigation is warranted.

From a mechanical perspective, the current analysis was restricted to two discrete phases of the gait cycle: heel contact at 25% and toe-off at 55%. However, in clinical scenarios, transfemoral prostheses are subjected to continuous, dynamic, and multi-directional loading throughout the entire gait cycle. This includes vertical compressive forces during the stance phase and shear forces during the swing phase. To more accurately represent the mechanical demands experienced *in vivo*, future studies should incorporate full gait cycle simulations to enable a comprehensive evaluation of load transfer characteristics and structural response.

In terms of experimental validation, the finite element results require confirmation through both *in vitro* and *in vivo* investigations. Mechanical testing of bone–prosthesis constructs, particularly under cyclic loading conditions, would yield essential data to corroborate the stress distribution and deformation patterns observed in simulation. Additionally, *in vivo* studies using appropriate animal models are necessary to evaluate critical parameters such as bone–prosthesis contact area ratio and interfacial micromotion, both of which are directly related to the success of long-term osseointegration.

From a biological perspective, the long-term biocompatibility of Mo–Re alloys, their ion release profile, and their potential effects on osteoblastic differentiation remain insufficiently characterized. Accordingly, further studies are required to evaluate the biological safety of Mo–Re alloys. These should include *in vitro* cellular assays, such as cytotoxicity assessments and measurements of alkaline phosphatase activity, as well as *in vivo* implantation studies focused on evaluating inflammatory responses, fibrous capsule formation, and tissue integration. Such investigations will be essential for establishing the biological safety and overall suitability of Mo–Re alloys for orthopedic applications.

## 5 Conclusion

In this study, four transfemoral prosthetic designs were developed, and their mechanical performance was evaluated through finite element simulations under physiological loading conditions corresponding to 25% (heel contact) and 55% (toe-off) phases of the gait cycle. The objective was to optimize prosthetic design and reliability by analyzing stress distribution patterns within the bone–prosthesis system.

Under the 25% loading condition, all four prostheses exhibited similar stress concentrations at the distal stem end, with maximum von Mises stress values ranging from 179 to 185 MPa under identical loading conditions. However, the porous prosthesis designs effectively avoided the inner-wall stress concentrations within the medullary cavity that were observed in the grooved prosthesis. Both porous designs demonstrated comparable load transfer characteristics, with the surrounding bone exhibiting a maximum von Mises stress of 20.4 MPa—within the “favorable stress window” for bone remodeling, as defined by established mechanobiological thresholds.

Furthermore, the Mo–Re alloy prosthesis, owing to its higher elastic modulus, exhibited reduced deformation under the same loading conditions. The maximum stress within the porous region of the Mo–Re prosthesis was 5.3 MPa, compared to 9.3 MPa in the titanium alloy porous prosthesis, thereby lowering the mechanical burden at the implant–bone interface.

In summary, the Mo–Re alloy porous prosthesis demonstrated favorable stress distribution characteristics, reduced porous-region stress, and minimized stress concentrations—mechanical advantages that may translate into improved clinical outcomes. These include enhanced load transfer, reduced risk of implant loosening due to lower interfacial stress, improved fatigue resistance, and greater potential for long-term osseointegration. Finite element analysis supported the feasibility of the Mo–Re alloy porous prosthesis for bone implantation; however, further experimental validation is required. Mechanical testing and *in vitro* and *in vivo* biological evaluations will be essential for confirming clinical applicability.

This study provides a biomechanical foundation for the iterative design of porous Mo–Re alloy prostheses aimed at maximizing mechanical compatibility. Future optimization efforts should focus on tailoring porous parameters (e.g., porosity gradients and pore size distribution) and refining stem morphology to match the material characteristics of Mo–Re alloys. Such integration of material, structural, and mechanical factors may enhance prosthesis stability and clinical performance while supporting patient-centered outcomes.

## Data Availability

The original contributions presented in the study are included in the article/Supplementary Material, further inquiries can be directed to the corresponding author.
