# Supplementary figures and images for: Finite element analysis of the biomechanical behavior of four osseointegrated prosthetic designs
Source: Front Bioeng Biotechnol. 2025 Dec 10;13:1694169. doi: 10.3389/fbioe.2025.1694169 (PMC12727996; doi:10.3389/fbioe.2025.1694169)

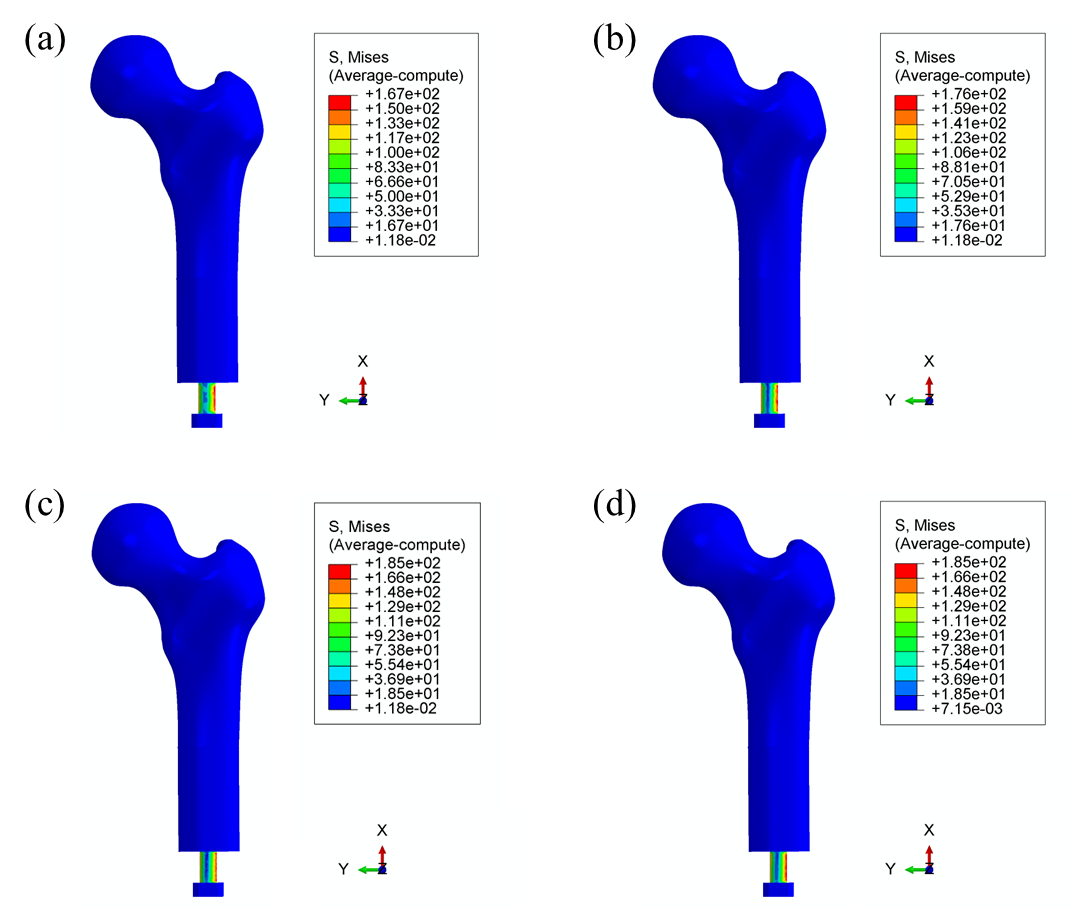

Supplement: Supplementary file 1 [file Image2.tif]

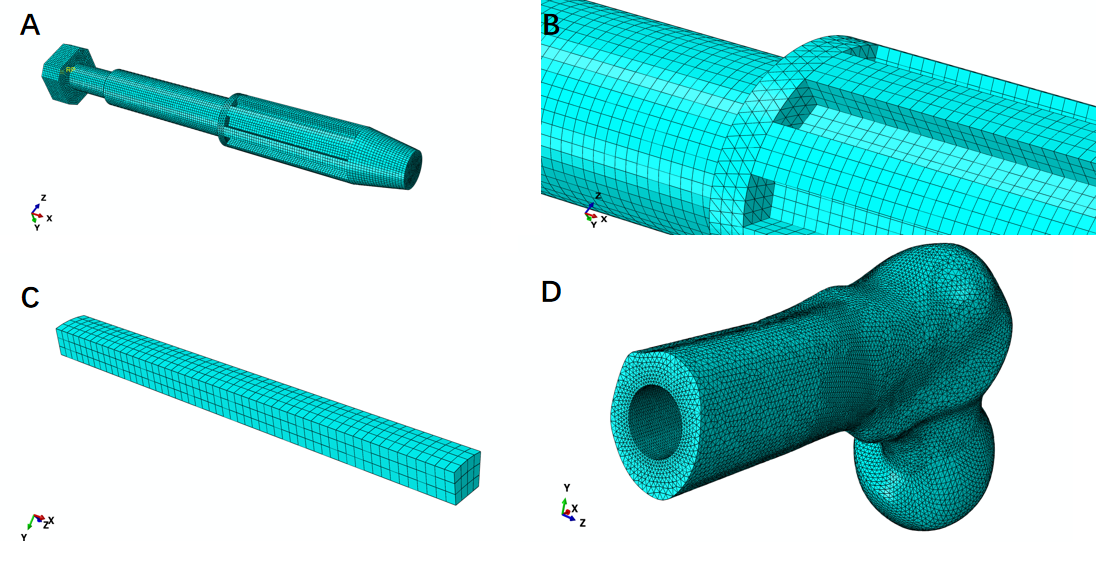

Supplement: Supplementary file 2 [file Image1.tif]
